# Primary Cutaneous Actinomycosis: A Diagnostic Enigma

**DOI:** 10.7759/cureus.37261

**Published:** 2023-04-07

**Authors:** Sulhera Khan, Bareerah Khan, Wajeeha Batool, Marium Khan, Amir H Khan

**Affiliations:** 1 Dermatology, Civil Hospital Karachi, Karachi, PAK; 2 Internal Medicine, Dow University of Health Sciences, Civil Hospital Karachi, Karachi, PAK; 3 Internal Medicine, Jinnah Postgraduate Medical Centre, Karachi, PAK

**Keywords:** madura foot, tuberculosis, infectious disorders, misdiagnosis, primary cutaneous actinomycosis

## Abstract

*Actinomyces* are Gram-positive, filamentous rods found endogenously as a part of the normal flora and can be acquired exogenously as they are present in the soil. The most common species known to infect humans is *Actinomyces israelii*. Five forms of the disease have been identified so far, of which the primary infection of the skin is the most uncommon. It is also commonly considered one of the most misdiagnosed diseases. We present a case of a young male diagnosed with primary cutaneous actinomycosis based on a histopathology specimen after multiple failed diagnoses of Madura foot/mycetoma, cutaneous tuberculosis, and malignancy. The patient was successfully treated with antibiotics with the restoration of his functional disability caused by the lesion.

## Introduction

Actinomycosis is a bacterial infection caused by a Gram-positive, filamentous bacteria called *Actinomyces israelii* [1]. The bacterium is a part of the normal flora of the mouth and colon but also causes opportunistic infections [1]. The pathogenic species are also found in the soil and can be acquired exogenously [2]. Six species of the bacterium are known to infect humans [1]. Apart from *Actinomyces israelii*, the other species include *Actinomyces bovis,* which commonly causes lumpy jaw in cattle and may also occasionally infect humans, *Actinomyces gerencseriae,* also known as *Actinomyces israelii *serovar II, *Actinomyces naeslundii*, which is known to cause periodontal disease,* Actinomyces radicidentis*, which is known to cause infected root canal abscesses, and *Actinomyces viscosus*, which has a low level of virulence [1]. Actinomycosis is known to cause chronic suppurative infection of various organs of the body with discharging sinuses and abscesses [3]. Five forms of actinomycosis have been identified in humans: cervicofacial, pelvic, abdominal, thoracic, and primary cutaneous [3]. Of the five subtypes, primary skin infection of the extremities is quite uncommon [4]. There is no specific data on the prevalence of actinomycosis worldwide and in developing countries like Pakistan.

Actinomycosis is one of the commonly misdiagnosed infections of the skin, and it can mimic tuberculosis, fungal infections, botryomycosis, and malignancy [2]. The diagnosis can be challenging and delay can result in significant progression of the disease and physical deformity [2]. We report a case of a laborer who was diagnosed with primary cutaneous actinomycosis based on histopathology and was successfully treated.

## Case presentation

A 22-year-old unmarried male, who was a resident of a rural area in Sindh, Pakistan, and a laborer by occupation presented to the outpatient department of a tertiary care hospital in Karachi, Pakistan with a complaint of a hard lesion on his left thumb that had developed over the last seven months. The lesion was painful with multiple discharging sinuses. The patient reported suffering an injury on his left thumb seven months back while lifting heavy bricks at work. The injury had gone unnoticed; however, after one to two weeks, the patient had developed swelling around the wound, which had been painless and gradually increasing in size. After two months, the swelling had developed into a hard nodular lesion on his left thumb, gradually progressing to involve the left index finger. Initially, the lesions had been painless but had gradually become painful and started bursting and discharging sinuses, revealing frank pus and occasionally bloody discharge. The patient also gave a history of yellow granules coming out with the discharging sinuses. The patient denied any fever, weight loss, or any other systemic symptoms. His past medical, surgical, travel, and sexual history was non-significant.

**Figure 1:**
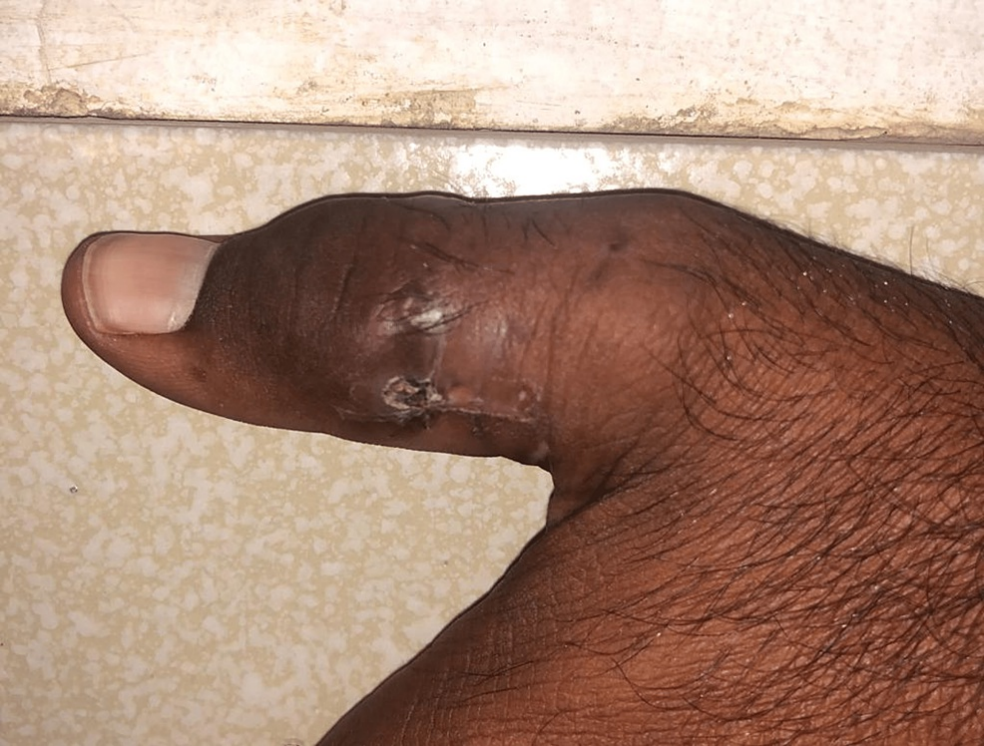
Image showing multiple lesions with erosion along the medial border of the left thumb

**Figure 2:**
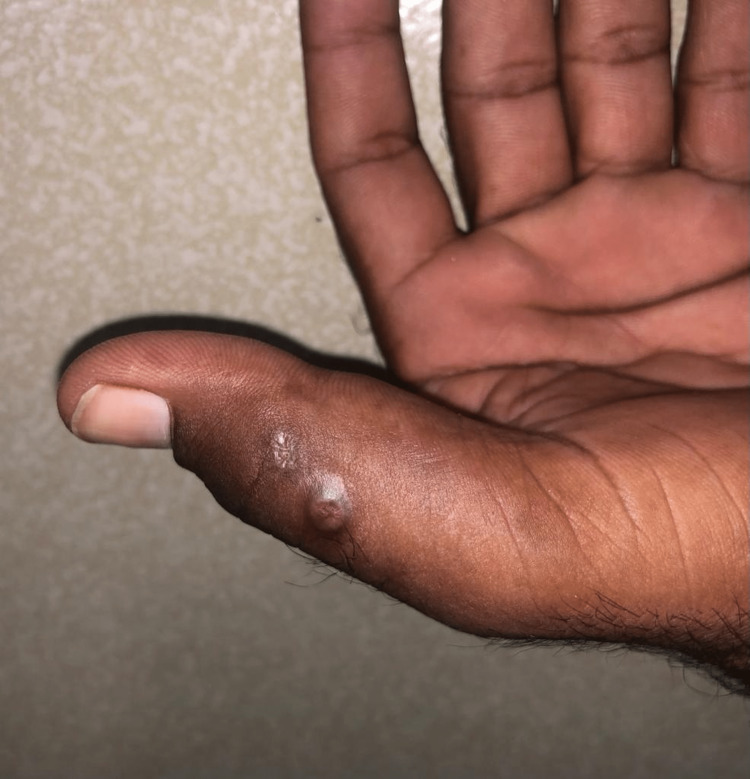
Image showing a single papule with central punctum and an atrophic depressed scar along the lateral border of the left thumb

On examination, the patient was found to be a young male of average height and build, who was alert and oriented; his vitals showed a blood pressure of 120/90 mmHg, a pulse of 87 beats/minute, a respiratory rate of 14 breaths/minute, and he was afebrile. On cutaneous examination, there was an ill-defined nodular swelling with discharging sinuses and overlying scarring and hyperpigmentation along the medial and lateral border of the left thumb and index finger (Figures 1, 2). However, no discharge could be appreciated from the sinuses. On palpation, the skin was hard, non-tender, fixed to the underlying structures, and overlying adherent skin. The satellite lesions were non-tender. The lesions were not warm. The cardiorespiratory, neurological, and abdominal examinations were normal.

The laboratory investigations of the patient were normal and are summarized in Table 1.

**Table 1:** Laboratory investigations during the hospital stay MCV: mean corpuscular volume; TLC: total leukocyte count; ALT: alanine transaminase; ALP: alkaline phosphatase; TSH: thyroid stimulating hormone; HBsAg: hepatitis B surface antigen; Anti-HCV: hepatitis C antibody; HIV: human immunodeficiency virus

Parameter	Day 1	Normal values
Hemoglobin (g/dl)	13.3	13.2-16.6 g/dl
MCV (fl)	82	80-100 fl
Platelets (x 10^9^/L)	178	150-450 x 10^9^/L
TLC (x 10^9^/L)	5.6	4.5-11 x 10^9^/L
Bilirubin (mg/dl)	0.6	0.1-1.2 mg/dl
ALT (U/L)	8	4-36 U/L
ALP (IU/L)	23	44-147 IU/L
International normalized ratio	1.0	<1.1
Creatinine (mg/dl)	0.7	0.7-1.3 mg/dl
Urea (mg/dl)	12	6-24 mg/dl
Sodium (mEq/L)	136	135-145 mEq/L
Potassium (mEq/L)	3.4	3.6-5.2 mEq/L
Chloride (mEq/L)	101	96-106 mEq/L
Bicarbonate (mEq/L)	24	22-29 mEq/L
Erythrocyte sedimentation rate (mm/hr)	4	0-22 mm/hr
C-reactive protien (mg/dl)	0.2	<0.9 mg/dl
Vitamin B12 (pg/mL)	650	160-950 pg/mL
Folate (ng/mL)	5.8	2.7-17 ng/mL
TSH	1.5	0.5-5 mIU/L
HBsAg	Non-reactive	
Anti-HCV	Non-reactive	
HIV antibody testing	Non-reactive	

**Figure 3:**
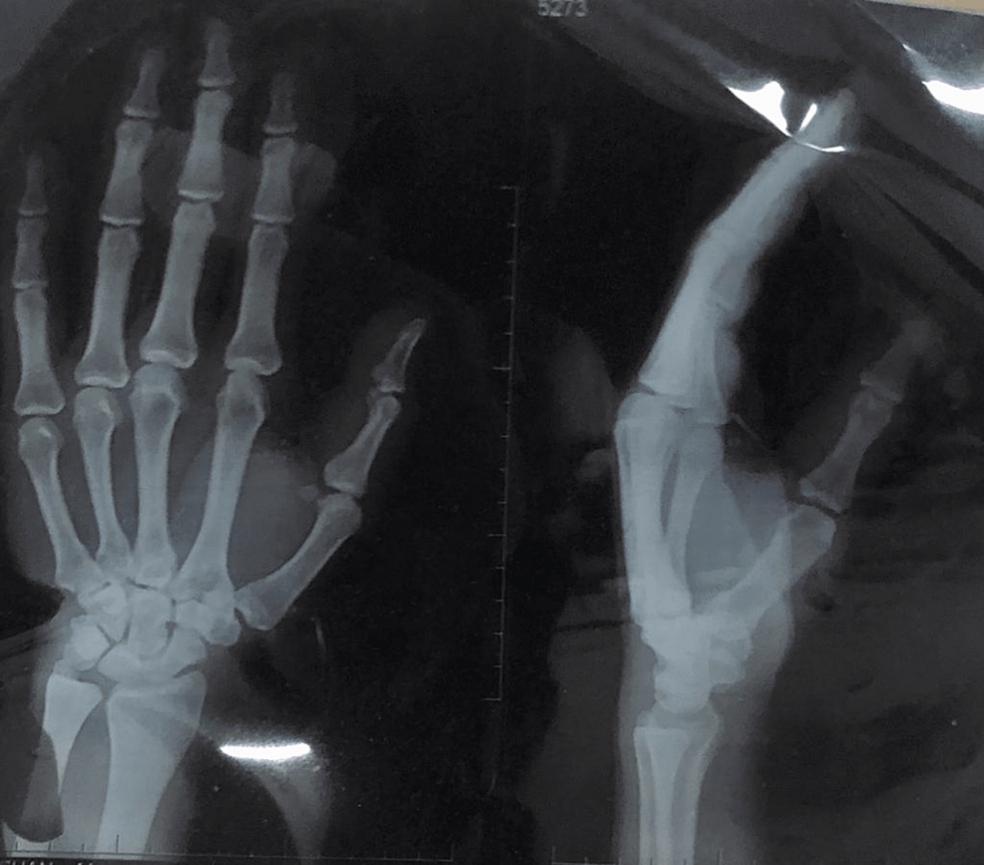
X-ray hand anteroposterior and lateral views showing no bony involvement with mild soft tissue swelling of the left thumb

**Figure 4:**
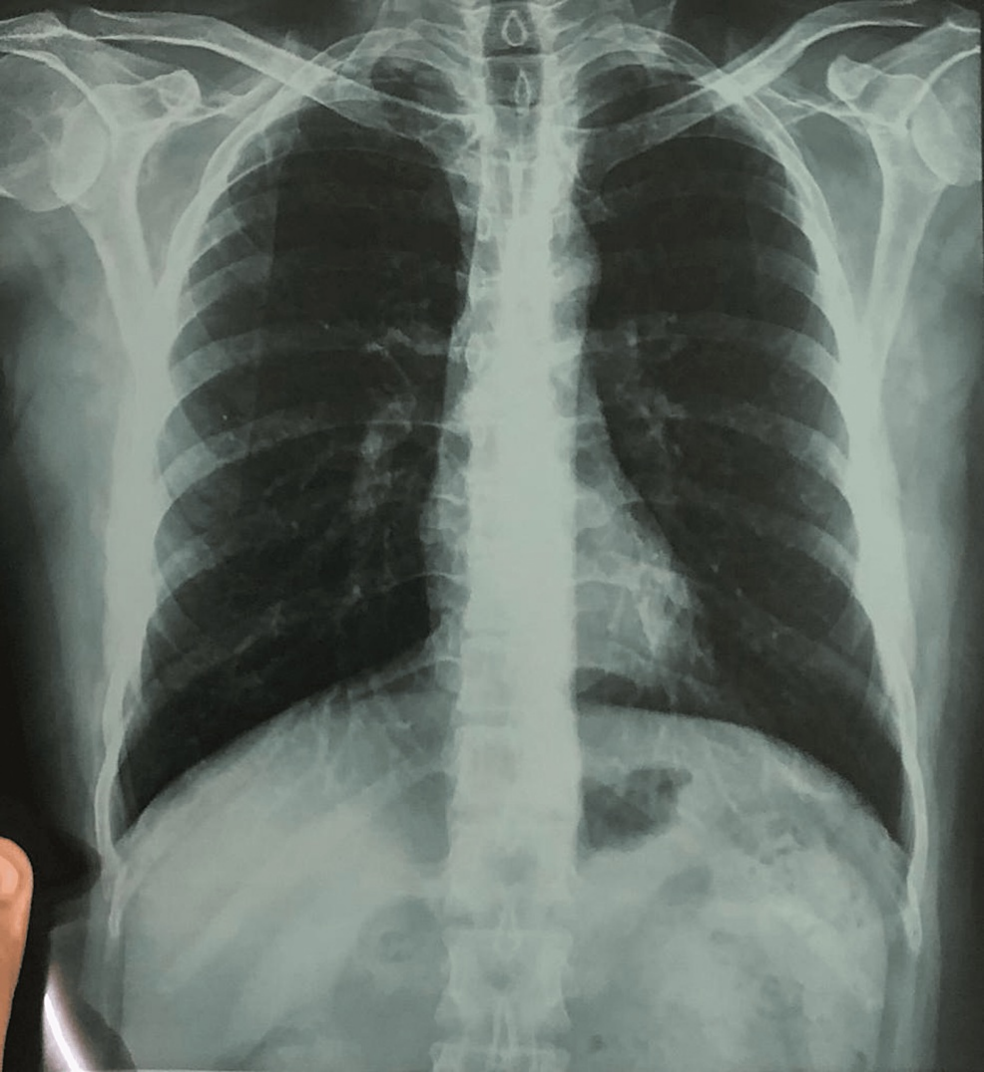
X-ray chest posteroanterior view showing a centrally placed trachea, normal cardiothoracic shadow, no mediastinal widening, and no osteopenia, masses, or infiltrates

X-rays of the left hand and X-ray chest are shown in Figures 3-4.

Initially, the patient was treated with oral (clindamycin) and intravenous (vancomycin) antibiotics, but no response was documented. The acid-fast stain was negative for tuberculosis. The acid-fast bacillus culture testing was also negative. To rule out malignancy and tuberculosis, a soft tissue biopsy was done from the left thumb, which revealed tissue lined by epidermis showing pseudoepitheliomatous hyperplasia and hyper ortho- and parakeratosis. The papillary and reticular dermis showed moderate acute and chronic inflammation and granulation tissue, also showing occasional bacterial colonies of a filamentous organism with characteristic neutrophilic outlining (Splendore-Hoeppli phenomenon). There was no evidence of granuloma formation and malignancy. Special stain periodic acid-Schiff (PAS) was negative for fungus. The culture of the specimen was negative, which is possible in cases with *Actinomyces* infection due to previous antibiotic therapies, inadequate culture conditions, or inadequate short-term culture. These findings were consistent with a cutaneous actinomycosis infection. The biopsy report differentiated actinomycosis from cutaneous tuberculosis, botryomycosis, malignancy, and bacterial abscess. The culture of the tissue could not be performed due to the patient's financial limitations. Pus could not be collected from the sinuses as they had stopped discharging and hence no granules could be appreciated.

As we were initially considering the diagnosis of actinomycosis versus nocardiosis, we had planned for concomitant therapy with both drugs until the biopsy and culture report arrived. As soon as the report came, the patient was started solely on penicillin, which showed a positive response, suggesting primary infection with *Actinomyces*. The patient was started on injection of benzylpenicillin 8 million units six hourly daily. He was then shifted to oral amoxicillin 500 mg every six hours. At the follow-up, the patient's lesions were found to have healed significantly with no draining sinuses and fibrosis.

## Discussion

*Actinomyces* is an endogenous organism in the mouth, intestine, and colon [5]. Due to the organism's endogenous nature, the skin's primary infection is seen to be very uncommon [5]. However, the bacterium can be acquired through trauma, perforating wounds, and compound fractures causing dermatological infection [5]. The bacterium is thought to cause the destruction of the local tissues via contiguous and hematogenous spread [5]. *Actinomyces* infections caused by insect bites have also been reported in some cases [6]. It can also be acquired secondary to thorn pricks [7]. In our patient, the cutaneous infection resulted from trauma during work at a construction site. Similarly, one study has reported an electric technician acquiring cutaneous actinomycosis during his work after a traumatic injury that went unnoticed [6].

Primary cutaneous actinomycosis is identified as one of the most misdiagnosed diseases [3]. The diagnosis of cutaneous actinomycosis is a dilemma as the disease mimics the clinical features of many other illnesses [4]. The disease needs to be differentiated from cutaneous tuberculosis, malignancy, botryomycosis, nocardiosis, and sporotrichosis [5]. Our patient was initially managed with oral and intravenous antibiotics with no resolution of symptoms and with further progression of the cutaneous disease. He gave a history of the presence of yellow granules, and, keeping in mind the concerns for mycetoma, fungal scrapings and culture were sent, which returned negative. Later on, in view of the suspicion of cutaneous tuberculosis, a soft tissue biopsy was sought for acid-fast bacilli culture and a GeneXpert test, which also came back negative. The biopsy report was sought, which gave the diagnosis of primary cutaneous actinomycosis. The sulfur granules had dried up and the absence of discharge precluded the identification of the Gram-positive filamentous colonies. *Nocardia* can be differentiated from *Actinomyces* as the former organism is weakly acid-fast [5].

For the successful eradication of the microorganism, surgical debridement with the appropriate choice of antimicrobial therapy, dosage, and duration is required [4]. Penicillin is identified as the initial choice of antibiotic for cutaneous actinomycosis for a treatment duration of six months to one year [4]. Hypersensitivity to penicillin requires treatment with clindamycin, tetracycline, erythromycin, doxycycline, and chloramphenicol [8]. The patients should also be counseled regarding the side effects and risks of long-term antibiotic therapy such as pseudomembranous colitis due to *Clostridium difficile* infection, interstitial nephritis, gastrointestinal symptoms such as epigastric pain and discomfort, and hematological abnormalities such as eosinophilia and leukopenia, predisposing to superinfection [4].

## Conclusions

Primary cutaneous actinomycosis is a rare entity mimicking other infectious and non-infectious diseases due to similarities in clinical presentations. Therefore, it is important to consider actinomycosis as a differential in patients with nodular swelling and discharging sinuses. It is also imperative to treat these patients with an appropriate antibiotic regimen for an adequate duration.
